# Understanding the attitudes and acceptability of extra-genital Chlamydia testing in young women: evaluation of a feasibility study

**DOI:** 10.1186/s12889-019-7313-0

**Published:** 2019-07-24

**Authors:** Sally Brown, Charlotte Paterson, Nadine Dougall, Sharon Cameron, Nick Wheelhouse

**Affiliations:** 1 School of Applied Sciences, Edinburgh Napier University, Sighthill Court, Edinburgh, EH11 4BN UK; 2 School of Health and Social Care, Edinburgh Napier University, Sighthill Court, Edinburgh, EH11 4BN UK; 3 Chalmers Centre, 2a Chalmers Street, Edinburgh, EH3 9ES UK

**Keywords:** Chlamydia trachomatis, Extra-genital, Sexual health, Women, Screening, Self-sampling

## Abstract

**Background:**

*Chlamydia trachomatis (C. trachomatis)* is the most common bacterial sexually transmitted infection in the UK. Recent studies suggest that in addition to the genital tract, *C. trachomatis* is found in the throat and rectum, suggesting the number of infections is under-reported. There is an urgent need to study the impact of extending diagnosis to include extra-genital samples; however, there is a lack of evidence on the acceptability of asking young women to provide these samples.

**Method:**

A mixed methods single group feasibility study explored the acceptability of combined genital and extra-genital testing in young women aged 16–25 years consecutively attending a sexual health centre in Edinburgh, Scotland. Young women were asked to complete a self- administered anonymous questionnaire whether they would be willing to give self-taken throat and ano-rectal samples. Interviews with women (*n* = 20) willing to self-sample were conducted before and after self-sampling, and these explored the underlying reasons behind their decision, and feelings about the tests.

**Results:**

Of 500 women recruited to the study, 422 (84.4%) women provided sufficient data for analysis. From completed questionnaires, 86.3% of respondents reported willingness to self-sample from the throat. Willingness of ano-rectal self-sampling was lower (59.1%), particularly in women under 20 (< 20 years: 44.4%; ≥20 years, 68.2%). Willingness of ano-rectal self-sampling was higher in women who had more sexual partners in the last 6 months (0 partners, 48.3%, *n* = 14, 3 or more partners, 67.4%, *n* = 60) and in those who have previous experience of a positive test for a sexually transmitted infection (STI) (positive: 64.5%; negative: 57%). Interviewed women suggested that a lack of knowledge of STIs, embarrassment and lack of confidence in the ability to carry out the sampling were barriers towards acceptability.

**Conclusions:**

In this study, self-sampling of throat samples is largely acceptable; however, the acceptability of taking an ano-rectal sample for *C. trachomatis* testing in young women was lower in younger women. The study suggests further research to investigate the acceptability of extra-genital testing as an addition to routine *C. trachomatis* testing, and whether this increases detection and prevents infective sequelae for women.

**Electronic supplementary material:**

The online version of this article (10.1186/s12889-019-7313-0) contains supplementary material, which is available to authorized users.

## Background

*Chlamydia trachomatis* (*C. trachomatis*) is the single most commonly diagnosed sexually transmitted bacterial infection in the UK, with over 203, 000 cases in England [1], and a further 15,685 cases in Scotland diagnosed in 2017 [2]. Young people are particularly at risk, with 67% of diagnosed cases in patients under the age of 25, equivalent to a detection rate of 1,882 per 100,000 population within this cohort [1]. However, many cases of *C. trachomatis* infection are clinically asymptomatic [3] and remain undiagnosed, and as a consequence the actual prevalence is likely to be higher. A recent Health Technology Assessment report highlighted a 17% risk of pelvic inflammatory disease (PID) after *C. trachomatis* infection is untreated in women by the age of 44 [4], with costs to the NHS of treatment of *C. trachomatis* infections and subsequent complications estimated at between £37–£412 (approximately $57–$536) per case [5]. Current diagnostic protocols of *C. trachomatis* infection in women involve sampling genital tract samples and subsequent analysis by nucleic acid amplification test (NAAT). These NAAT’s are validated for vulvo-vaginal samples but they are required to be validated locally before they are used routinely [6]. Systematic analysis of recent international clinical studies suggest that between 5 and 30% of infected women carry extra-genital chlamydial infections which sampling the genital tract alone would fail to identify [7]_,_ suggesting that combined genital/ extra-genital testing regimens will increase detection sensitivity. Extra-genital infections are also of clinical significance. Rectal infections appear to be associated with Azithromycin treatment failure [8], provide a reservoir for re-infection of the genital tract, and repeat re-infection is associated with increased risk of complications such as PID.

In women there is no clear association between receptive anal sex and rectal chlamydial diagnosis unlike in men who have sex with men (MSM) where targeted diagnostic protocols are effective. The recent British Association for Sexual Health and HIV (BASHH) national guidelines for the management of infection with *C. trachomatis* conclude that ‘Further studies with larger numbers of patients are needed to ascertain the utility of targeted versus routine rectal sampling in women’ [9]. However, the cited evidence for favourable acceptability of self-taken rectal and pharyngeal swabs in this report was restricted to studies on MSM [9], a consequence of the poor existing published data on the acceptability of extra-genital testing in women, in the UK. While there has been no published work in this area from the UK a study of women attending an STI clinic in the Netherlands found that 94% (950 of 1012) were willing to undertake extragenital testing [10].

It is well established that sexually transmitted infections are associated with stigma [11], and that attending a sexual health clinic may also be perceived as stigmatising and embarrassing [12]. While there are many negative implications of a *C. trachomatis* diagnosis, including shame, embarrassment and worry [13], Balfe et al. [14] found that young women would be prepared to accept opportunistic screening for chlamydia despite fearing that their identities would be stigmatised, as they perceived screening as a responsible act. Normalising screening by offering it to everyone, rather than identifying some people as ‘needing’ screening, would increase acceptability [15].

The primary aim of this study was therefore to assess the attitudes towards extra-genital testing of young women undergoing STI testing using a mixed methods approach of a survey and qualitative interviews.

## Methods

### Study design

This is a nonrandomised single group feasibility study [16]. A mixed methods design was used to address the following objectives: (1) What are young women’s attitudes to being tested for extra-genital *C. trachomatis*; (2) Does proposed willingness to complete self-sampling differ on socio-demographic factors, specifically age (women < 20 years of age compared to women aged 20–25), ethnicity, education level, deprivation level, smoking, sexuality, number of sexual partners and self-reported previous STI positivity; (3) What are women’s attitudes to diagnostic protocols including extra-genital testing in routine practice; Ethical approval was provided by Edinburgh Napier University and NRES committee North West- Preston (REC reference: 17/NW/0396).

### Participants, setting and data collection

Women aged between 16 and 25 years old consecutively attending a Sexual Health Centre in Edinburgh were deemed eligible. The Sexual Health Centre provides a range of sexual and reproductive health services to Edinburgh and the Lothians with a population of approximately 800, 000. People are referred by primary care services or can self-refer via any of the drop-in clinics. Approximately 500 women per month under the age of 20 routinely undergo Chlamydia testing at the Sexual Health centre. Women who attended the clinics were invited to complete a bespoke study questionnaire by the receptionist.

If willing to take part, women were asked to complete a bespoke quantitative questionnaire collecting information regarding willingness to complete self-taken throat and ano-rectal swabs, sexual activity, use of contraception, sexuality, previous STI testing (Additional file 1). Demographic information (age, ethnicity, education, and smoking) were also collected. A clinical research nurse was on site to answer women’s questions. Completed questionnaires were placed in an opaque envelope in a sealed box. Women completed as much or as little as they wished, and this did not influence their clinical care. Consent for use of questionnaire data was implied by completion. Prior to commencing the full study, the questionnaire was piloted with 20 young women (aged 16–20 years) and appropriate amendments were made to improve question clarity.

The final question of the questionnaire asked whether women would take part in an interview about their attitudes towards extra-genital testing. Participants were offered a £10 voucher as in lieu of travel expenses for taking part in the qualitative interview. Those who wished to do so completed a tear off slip with their name and contact number and placed that in a separate opaque envelope, so that the research nurse could contact them to arrange a date for interview with the qualitative researcher. Once the slip had been removed from the questionnaire, the personal contact information could not be linked to data provided in the questionnaire, which remained anonymous.

To address objectives one and three, we aimed to carry out structured qualitative interviews with women who were and were not willing to carry out extra-genital testing (see Additional file 2 for the interview topic guide). Informed written consent was obtained to participate in recorded qualitative interviews. Twenty women who were willing to carry out the test agreed to be interviewed. They attended the clinic and had a short interview about how they felt about testing, were then given the testing kits by a research nurse and took a throat and rectal swab themselves and were then interviewed about the procedure. Pre- and post-test interviews were conducted by two of the research team (SB and CP). Although the interviews took place in a clinic setting, the participants were informed during the consent process that the interviewers were university rather than clinic staff; we made the decision that the research nurse would not conduct any of the interviews as it was felt that participants could be less likely to report any negative feelings to her. However, although the setting itself may have meant participants were more likely to report positive views about the tests, many did discuss what they felt were negative aspects of the testing. No women who had indicated that they would not be willing to do the tests volunteered to be interviewed.

### Sample size

As recruitment rates were initially unknown, sample size estimation was a two stage process. Firstly, using a 99% confidence level (margin of error + or – 5%) to detect 50% acceptability (i.e. 45–55%); with an estimated recruitment rate of 70%, an estimated 952 women were required to be approached to participate. From the first 250 women consented, the sample size was re-estimated. Using a 95% confidence level (margin of error + or – 5%), to detect a 60% (i.e. 55 to 65%) acceptability and an estimated recruitment rate of 70% (based on the recruitment and acceptability rate at this stage) we aimed to consecutively approach 528 women. We aimed to recruit for qualitative interviews, sub-samples of women who were (*n* = 20) and were not (*n* = 20) willing to carry out extra-genital testing, to understand reasons for accepting or declining the test.

### Analysis

Quantitative data were analysed using IBM SPSS 23. Demographic and willingness responses and recruitment rate (i.e. proportion of those who consented as a percentage of those who were invited to participate) were presented as descriptive summaries for all participants and for sub-groups of women (aged < 20 and > or = to 20 years). Specifically, means and standard deviations, or medians and IQR were reported for continuous data where appropriate, and count data and proportions were reported for categorical data. Where proposed willingness varied between subgroup categories, post-hoc analyses were conducted using chi-square test of independence.

The qualitative interviews were recorded with the consent of participants, and fully transcribed. Transcripts were analysed by two of the research team (SB and CP) using a Framework Analysis approach [17], which is appropriate for analysing qualitative data in a multidisciplinary health-related project [18]. Framework Analysis has five steps: familiarisation, identifying the framework, indexing, charting, and mapping and interpretation. SB and CP first familiarised themselves with the data by reading all the transcripts independently, noting what seemed to be the emerging themes. We then met to jointly review the transcripts and our initial thoughts about the coding framework. As the study was designed around a specific set of issues, this guided the development of the framework, but we also incorporated novel themes, thus using both deductive and inductive approaches to ensure we did not miss any important themes. Having developed our initial framework, we again reviewed the transcripts independently before meeting to compare our indexing and chart the data. Any disagreements about coding were resolved through discussions at these meetings. Having reached agreement about the final indexing and charting, we then mapped the data and worked on the interpretation together.

## Results

Results are summarised in two sections, firstly the results from the questionnaire survey, followed by the findings from the interviews. The interview findings are presented under the two key themes relating to acceptability that we drew from the analysis, feelings pre- and post-test, and where testing takes place.

### Sample description, recruitment rate, participation rate

**Fig. 1:**
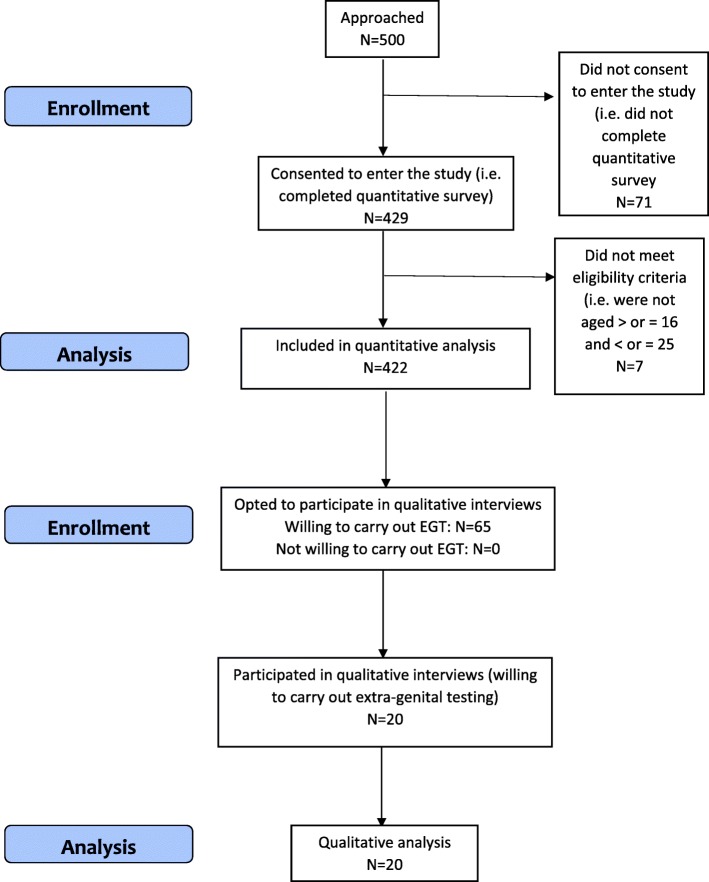
Flow-chart summarising study design and patient recruitment

During the study period, December 2018 to February 2019, 500 women were consecutively approached (i.e. given the information sheet and questionnaire). Of the 500 approached, 71 (14%) did not return forms and were therefore not included in the study. Seven patients (1.4%) returned the survey but were not eligible (i.e. did not meet the included age range) and were excluded from the analysis, leaving a final sample of 422 (84.4%) deemed to have consented and participated (see Fig. 1). The target sample (*n* = 528) was not met, however this target included a 30% increase to allow for refusal. The target sample size before accounting for a 70% recruitment rate (*n* = 369) was exceeded in reality (*n* = 422), therefore power was not compromised. Furthermore, when adjusted for the actual recruitment rate (84%), the estimated target sample size would be *n* = 439, which our sample exceeds. As 422 women were approached to participate over the course of 3 months, this implies that future work stemming from this epidemiological work should plan to approach 164 eligible women per month, to obtain a recruitment rate of 140 women per month. Participant characteristics are presented in Table 1. Women living in the most deprived areas are underrepresented in our sample (8%, *n* = 32), compared to the population residing in the council area of Edinburgh (13%, *n* = 59, 280) [20]. Less educated women are also underrepresented in our sample compared to the population residing in the catchment area of NHS Lothian [21] Of our sample, 97.5% had attained qualifications at level 4 or above (*n* = 412) and fewer had no qualifications (2.5%, *n* = 10) compared to NHS Lothian (33.7%, *n* = 233, 387, and 21%, *n* = 145, 434, respectively, [21]. There was also an overrepresentation of non-white women in our sample (9%, *n* = 40), compared to the population in the catchment area of NHS Lothian (5.6%, *n* = 46, 724) [21].

**Table 1:** Participant characteristics and reported willingness to self-sample

Parameter	Summary data	Percentage of women who reported willingness to complete self-sampled throat testing	Percentage of women who reported willingness to complete self-sampled extra-genital testing
Age (*n* = 422) (m, SD)	20.5 (2.5)	N/A	N/A
Age group (*n* = 422): n (%)			
Under 20 years	161 (38.2)	134 (83.2)	71 (44.4)
20 years and above	261 (61.8)	230 (88.1)	178 (68.2)
Total	422 (100)	N/A	N/A
Ethnicity (*n* = 422): n (%)			
White	380 (90.0)	329 (86.6)	224 (59.1)
Self-identified as non-white	42 (10)	35 (83.3)	25 (59.5)
Total	422 (100)	N/A	N/A
Sexuality (*n* = 422): n(%)			
Heterosexual	352 (83.4)	311 (88.4)	205 (58.4)
Other (Bisexual, prefer to self-describe, gay, bisexual, prefer not to say)	70 (16.6)	53 (75.7)	44 (62.9)
Total	422 (100)	N/A	N/A
Education (*n* = 408): n (%)			
No qualifications	9 (2.2)	7 (77.8)	5 (55.6)
Secondary education (compulsory)^b^	37(9.1)	28 (75.5)	13 (35.1)
Upper secondary education (optional)^c^	221 (54.2)	195 (88.2)	129 (58.6)
Vocational qualification (including SCQF levels 4, 5, 6, 8 and 11)	18 (4.4)	13 (72.2)	9 (50.0)
Further education^d^	118 (28.9)	105 (89.0)	81 (68.6)
Higher education^e^	9 (2)	5 (100)	0 (0.00)
Total	408 (100)	N/A	N/A
Deprivation level^a^ (*n* = 367): n(%)			
Affluent	71 (19.3)	65 (91.5)	45 (63.4)
Intermediate	183 (49.9)	150 (82.0)	105 (57.4)
Deprived	16 (4.4)	14 (87.5)	7 (43.8)
Very deprived	97 (26.4)	85 (87.6)	61 (62.8)
Total	367 (100)	N/A	N/A
Smokers (*n* = 416): n (%)			
Yes	110 (26.4%)	90 (81.8)	65 (59.1)
No	306 (74.6)	244 (87.5)	164 (59.0)
Total	416 (100)	N/A	N/A
Previously tested positive for an STI (*n* = 422): n (%)			
Yes	121 (28.7)	114 (94.1)	78 (64.5)
No	301 (71.3)	250 (83.1)	171 (57.0)
Total	422 (100)	N/A	N/A

Not Applicable (N/A)

^a^Deprivation level as measured using Depcat score which is based on the Carstiars & Morris index of deprivation and has been widely used in Scotland. Edinburgh is located in the health board catchment area of NHS Lothian [19]

^b^Standard grades, Intermediate grades 1 or 2; General Certificate of Secondary Education level 4;

^c^Scottish Credit and Qualification Framework level 6–7; Higher and Advanced Higher Grades;

^d^Higher National Certificate;Higher National Diploma

^e^Batchelor degrees and other postgraduate degrees

Data also revealed that willingness to self-sample did not appear to be affected by education, deprivation or ethnicity. For example, the proportions were broadly similar for the summary data and those willing to self-sample for education (across the columns lower educational attainment was 70.0% (*n* = 285/408), 68.8% (*n* = 243/353) and 65.8% (*n* = 156/237) respectively); for less deprived areas (69.2, 68.5 and 68.8% respectively); and regarding self as ‘white’ (90.0, 90.4 and 90.0% respectively, all Table 1).

### Data quality

Out of range responses were rare. Eighty (19%) participants had at least one missing data point, however this only amounted to 1.6% missing data overall. Of the 19%, 55 participants (13%) did not complete a valid postcode, suggesting that the missing data for postcode was not missing at random, either participants did not know their postcode or were less willing to complete this question. As there was still less than 2% missingness overall, no data imputation methods were used.

### Quantitative acceptability response

More women reported willingness to self-sample a throat swab (*n* = 364, 86.3%) than an ano-rectal swab (*n* = 249, 59.1%). Of those who reported unwillingness to self-sample a throat swab, 9% (*n* = 40) expressed concern about taking the sample from the wrong place, 7% (*n* = 28) indicated wanting a healthcare professional to take the sample, and 2% (*n* = 8) said they did not want anything in the throat. Whether women had a previous experience of testing positive for an STI was statistically significantly associated with increased willingness from women who had previously tested positive (see Table 2). Sexuality was also statistically significantly associated with increased willingness to self-sample throat swabs, however reported willingness tended remain high (> 70%) across subgroups (see Table 2).

**Table 2:** Associations of perceived willingness to self-sample throat swab

Variable		Willingness n(%)	Unwillingness n(%)	Unsure n(%)	Chi-square statistic (df)	*p*-value
Previous positive STI test (*n* = 422)	Yes n(%)	105 (94.1)	9 (7.3)^b^		9.11 (2)	0.01
	No n(%)	250 (83.1) 6 (75.0)	51 (16.9)^b^			
Sexuality (*n* = 422)	Heterosexual n(%)	311 (88.4)	16 (4.5)	25 (7.1)	7.97 (2)	0.02
	Other^a^ n(%)	53 (75.7)	6 (8.6)	11 (15.7)		

^a^(including bisexual, gay, lesbian, those who prefer to self-describe or not disclose)

^b^Aggregated with women who were unsure due to sparse data for some options

Of the 422 women, 59.1% (*n* = 249) reported proposed willingness to complete ano-rectal self-sampling,26.6% (*n* = 112) reported being unwilling and 14.3% (*n* = 60) were unsure. Eight percent (*n* = 33) of women expressed not wanting anything to touch the ‘back passage’ (anus), 8% (*n* = 33) expressed concern about taking the sample from the wrong place, 13% (*n* = 55) said they would prefer a healthcare professional to take the sample, and 16% (*n* = 69) said they did not want anything in the ‘back passage’ (anus).

Reported willingness to complete self-sampling of ano-rectal testing was higher for those who were older than 20 years (44.4%) compared with those under 20 years (68.2%); those who had sexual partners in the previous 6 months; and those who had previous experience of testing positive for an STI. However only age groups emerged as having a statistically significant association (see Table 3) with willingness to self-sample.

**Table 3:** Associations of perceived willingness to self-sample ano-rectal swabs

Variable		Willingness n(%)	Unwillingness n(%)	Unsure n(%)	Chi-square statistic (df)	*p*-value
Age (*n* = 421)	< 20 years n(%)	71 (44.4)	61 (38.1)	28 (17.5)	24.31 (2)	0.00
	20 years and above n(%)	178 (68.2)	51 (19.5)	32 (12.3)		
Number of sexual partners in the last 6 months (*n* = 418)	None n(%)	14 (48.3)	11 (37.9)	4 (13.8)	5.92 (6)	0.43
	1 n(%)	87 (60.8)	35 (24.5)	21 (14.7)		
	2–3 n(%)	87 (55.4)	45 (28.7)	25 (15.9)		
	More than 3 n(%)	60 (67.4)	20 (22.5)	9 (10.1)		
Previous positive STI test (*n* = 421)	Yes n(%)	78 (64.5)	21 (17.4)	22 (18.2)	8.48 (2)	0.08
	No n(%)	171 (57.0)	91 (30.3)	38 (12.7)		

### Qualitative results

The anonymised quotes below are identified by participant number and age (i.e. P1/23). The interviewer is indicated by ‘I’.

#### Feelings about the test

Almost all participants were unaware that the anus and throat could be a reservoir for infection, and were happy to do extra-genital testing if it gave more reliable results than solely vaginal testing.

P: *Obviously if it’s possible that you can have it without it being detected by the other swab it’s really good that it gets done in both areas* [throat and rectum] *now so I’m fine.*

*I: Great. And how are you feeling about doing the anal swab as well?*

*P: I’ve not done that one either. I guess I’m fine with it as well. Like they wouldn’t do it if it wasn’t for a purpose. So I guess if it means that it can protect people it’s a good things so I’m fine with doing it [laughs]. (P9/23)*

This participant appeared to have a reasonable understanding of STIs and their transmission, but felt that as others might not, expanding testing as well as raising awareness was necessary.

*I guess you can still have STDs without them coming up in the other tests. So it makes sense just to make sure that it’s not in any other places. I think it’s good cause a lot of people don’t realise you can get STDs even through just oral sex. So I think it’s quite good to raise awareness and also to pick it up as quickly as possible. (P9/23)*

Pre-test worries related to gagging while doing the throat swab and discomfort while doing the rectal swab:

*A little bit nervous about a throat swab just because like even brushing my teeth I usually make myself gag [laughs], so that’s just a little bit, I think it’ll be a bit tricky to do it. (P2/22)*

*Probably both of them will be uncomfortable but I don’t think it will be unbearable. (P14/19)*

There was also some uncertainty about whether they would do the ano-rectal test correctly as they had not done one before:

*And also, I suppose, would be a bit worried like how far do you need to put that in for it to be, you know, to work. Whereas, with the vaginal one, because like I regularly, well not regularly but I have, I have vaginal sex so like, you know, it can go up where I don’t regularly do it the other side. (P12/24)*

Specific worries relating to the ano-rectal swab related to embarrassment due to the part of the body being tested.

*I’d be a bit maybe embarrassed like if it came out and there was like a bit of poo on it or something. (P12/24)*

*It’s not like scared but I’m being shy yeah. (P16/25)*

However, despite worries about discomfort and ability to do the tests correctly, all the women went on to see the research nurse to be given the testing kits and instructions, and all but one did both tests, indicating that these worries were insufficient to deter them from taking the swabs. One participant changed her mind between the first interview and doing the test, deciding that she would only do the throat swab and not the ano-rectal swab.

*I’m just no too comfortable tae dae*^1^ *[pauses] things like that eh. So … I just didnae*^2^ *fancy doing it (P1/23)*

Once the women had completed the testing and given the samples to the research nurse, they returned for a short follow up interview. Acceptability of the tests was high, with all participants saying that they would be prepared to do the tests again, apart from the one woman who had changed her mind about the rectal swab but was happy to carry on doing throat swabs. All but this one participant suggested that they could become part of routine care. Indeed, one woman queried why they were not already part of routine care for women, citing a gay male friend who had told her that this type of testing was regarded as routine for his community.

*I was speaking to my friend who’s gay. And he told me that at the men who have sex with men clinic it’s just standard procedure. And we were saying, ‘well if it’s, if it’s correct that it can, chlamydia can live elsewhere in your throat or like anus, then why is it even a, why, why do you even have a choice kind of thing? Like if they, like why not just say, ‘you need these tests?’ (P11/24)*

There was a sense of resigned acceptance in that many of the women said they felt they should ‘just get on with it’, comparing the tests to injections, blood tests or cervical smears in terms of an experience that they felt was necessary despite it being unpleasant.

*I just felt like I had tae just dae it, just for precaution obviously (P1/23)*

*I just kind of think like no-one likes injections but everyone just deals with it. (P11/24)*

On the whole, participants who had expressed concerns in the first interview said that taking the swabs had not been as bad as they thought it would be; P7 had previously said she felt nervous about the rectal swab:

*P: It actually wasn’t as bad as I thought it would be. I was kind of, like, dreading the anal swab, but it actually wasn’t that bad.*

*I: What was it that you were dreading about it?*

*P: I don’t know, I just wasn’t sure if it was gonna be, like, uncomfortable, or anything, but it wasn’t, it was just kind of, I did it, it was over [laughs]. (P7/19)*

Some of the participants had found the throat swab made them gag, but apart from that it had not been a problem to do it; there were more practical challenges associated with the anorectal swab. Some had found the test awkward to do, partly because it had been difficult to find a comfortable position to insert the swab correctly, and partly because the swab itself was somewhat flimsy which had made inserting it difficult:

*I think the only thing with that one is that it was maybe a bit like flimsy. So I found trying to kinda guide, and I was sort of going, I was sort of using the mirror [both laugh] sort of, trying to sort of guide it in that way. I don’t know, maybe, it’d just be for me personally maybe one that was just a little bit like stiffer, I would have found more helpful*. *(P2/22)*

The concern that some participants had expressed prior to the tests, about whether they would do them correctly, was reiterated by several of them afterwards, with some uncertainty being expressed about whether they had done the tests correctly, partly because they had not done them before, with most of the reservations relating to the rectal test:

*P: The mouth one was easy, the other one was a bit more difficult cause I’d never had to do something like that [laughs] so but it was fine yeah.*

*I: How was it difficult?*

*P: Just because I wasn’t really used to, well sticking things [laughs] up there. (P9/23)*

*My only worry was, though, I still think, ‘did I do that right?’ (P12/ 24)*

Therefore, in terms of whether or not extra genital testing is acceptable, all but one of the women who returned to the clinic to do the tests said that they would accept them as part of routine care, that they understood how to do the tests, and found the instructions and the process to be clear. One was happy to do a throat swab but had changed her mind about the rectal swab and was uncertain about whether she would do the test at another time.

#### Location

Participants were asked how they felt about visiting a sexual health clinic, and for their views on the possibility of home testing. The key themes identified related to uncertainty and reassurance about the tests, and convenience versus stigma around attending a clinic.

Some participants felt that doing tests in a clinic meant they would be more reliable:

*I’d always be wary though ‘cause I’d feel like they wouldn’t be as accurate … it seems more official here. (P15/19)*

Although some liked the idea of home testing, they suggested that the clinic might be preferable if they felt unsure about how to do the test or whether they were doing it correctly, because there would be someone on hand that they could ask for guidance:

*I think [home testing] would like be good unless I had any questions because I knew that if I didn’t know what to do I could come out and ask.* *(P14/19)*

One participant suggested that home testing would be a good option if a woman had learnt how to do the tests at a clinic visit, and then felt confident they could do it correctly at home:

*I think it’s important to, for the first time you ever do it to come into the clinic because I really wouldn’t have known what I was doing. But I think once you’ve done it once in the clinic, I think having the option to do it at home if you’re pretty confident in what you can do.* *(P20/21)*

Some participants preferred to attend the clinic for testing partly because it would be more inconvenient for them to have to post samples back for testing than to do everything (be given the kits, do the swabs, hand them back) on one site. There was also some concern about samples getting lost in the post, or participants not knowing what had happened to them once they had been posted.

*I think I would prefer tae come in and just have it all set up instead a’ posting it. I think it’s a hassle tae post. I think it’s easier just to come in and have it all in the one place. … Yeah I think like I can just come in and everyone can just have the stuff there and give it to me. And I can just leave it there and you can give the results. But I think if you sent it out to me, I’d have to like go back, post it. I wouldn’t know where it’s going and things.* *(P13/21)*

The main reason for wishing for home testing to be an option was convenience.

*I think it would probably…like maybe make me more likely to do it. Like if I was really busy and I didn’t have time to do the commute both ways and be like wait to see somebody because obviously everybody here is really busy. So maybe if I didn’t have a lot of time it would be better to do it at home.* *(P14/19)*

This partly related to the clinic being in a city centre location, and services largely being centralised there so that for most people, accessing sexual health services means travelling in to the city centre.

An issue that was raised by several participants related to whether or not a visit to a sexual health clinic was stigmatising. Whilst some felt that it was potentially embarrassing to be seen entering the clinic, others suggested that attending sexual health services was becoming normalised:

*I mean sometimes people are warned off coming to these places because a’ the fear of someone they might see or, like, I have been in this situation where I’ve, I’ve taken my friend here and one of her past encounters was sitting with us [laughs]. So yeah I can see where it would be nice to have it at home and not have to deal with a whole situation.* *(P18/22)*

*I think it’s quite…very accepted nowadays in that, in like youth culture I guess. Like it’s not embarrassing to say that you’re going to the clinic even if people other than your friends overhear kind of thing. Like it’s very normalised. I don’t think there’s that much of a stigma around it. (P11/24)*

While this respondent felt that it was becoming accepted amongst younger age groups, another felt that people would feel less embarrassed as they got older:

*I think especially a lot of people, especially if they are younger get embarrassed about coming in. … I think it’s, I dunno, I think kind of like I’m at an age now I’m kind of like I don’t see it as big deal. But I do remember being like sixteen, seventeen and I’d always be like, ‘oh I don’t want anyone to see me go in’. And you’d be worried that everyone would be like, ‘oh they’ve got chlamydia’.* *(P2/22)*

Particularly where people are part of a small community where everyone is known to each other, such as a school, the assumptions made about attending the clinic meaning ‘you’ve got chlamydia’ could result in a stigmatised identity just by being seen at the clinic.

Therefore, in terms of whether or not visiting a clinic is acceptable, all the women felt that it was, although they also acknowledged the risk of it being a stigmatising encounter. Developing the option of doing these tests at home would also be desirable, although with the clinic was valued because of the advice and guidance available from the staff who were seen as friendly, helpful and non-judgemental.

## Discussion

This feasibility study has demonstrated that extra genital tract testing is largely acceptable in women aged 16–25 attending a sexual health clinic in Edinburgh, especially for self-taken throat swabs. Reported willingness to complete ano-rectal self-sampling was lower than that of throat swabs, but the acceptability of ano-rectal self-sampling was higher with increasing age. Specifically, just less than half of women under 20 years reported being willing to complete ano-rectal self-testing testing compared with two thirds of those women aged 20 to 25. Reported willingness to complete ano-rectal self-testing was also higher in women who had any sexual partners over the previous 6 months, and who had received a prior diagnosis of an STI. This suggests a greater willingness to undergo testing in women who perceive they have been more at risk of acquiring an STI [22].

Qualitative data highlight women’s pre-test concerns, primarily relating to discomfort or performing the test incorrectly and embarrassment. These concerns over the ability to conduct self-sample STI testing accurately by young people are consistent with previous observations [22]. After completing the tests, some women also reported uncertainty in relation to their own conduct of the self-tests and some discomfort, such as gagging during the throat swab and awkward positioning during the ano-rectal swab. Despite women having these concerns, it has been demonstrated that there is actually good concordance between the results gained from ano-rectal self-sampling with those obtained in a clinic [23]. It has been suggested to overcome the gagging response, that mouth rinses may be an alternative to throat swabs for both *Neisseria gonorrhoeae* and *C. trachomatis*, however this leads to an apparent reduction in sensitivity limiting its clinical effectiveness [24]. Importantly, in terms of clinical utility, women tended to report, post-test, that they would do it again, despite such uncertainty and/or discomfort, suggesting acceptability of including extra genital testing in routine diagnostic assessment procedures.

Qualitative data also suggested that women’s preference over location (in clinic or at home) and administrator (healthcare professional or self) of the tests differed. Such differences stemmed from women’s confidence in their ability to administer the test correctly or not, but also their perceived convenience of location. Women discussed stigmatisation as a reason for preferring home testing, with one participant suggesting this may be particularly true for younger people. Indeed, the shame associated with STI testing has been shown to be a significant barrier to testing [25]. However, in a recent study carried out in London, which compared the characteristics of people (men and women) undergoing online self-sample testing versus in clinic testing, 16–20 year olds were more likely to undergo testing within the clinic setting. Conversely, 20–25 years olds were more likely to use online self-sampling service [26]. This suggests that younger people who perhaps have not undergone testing previously may have more confidence in specialist expertise of clinical staff [27]. This may explain the differences in acceptability for women under (less acceptable) and over 20 years of age (more acceptable) found in the quantitative data. However, further investigation of why women under 20 years find extra-genital testing less acceptable is required.

### Strengths and limitations

This study is the only survey to date to assess the proposed willingness to complete self-sampling for extragenital *C. trachomatis*. The study had a good response rate and our mixed-method approach has provided additional information to that which a questionnaire-based study alone would have failed to obtain. However, there are limitations to the study. Within the survey, postcodes had the lowest completion rate (13%, *n* = 55). It is likely that this is not random and is indicative that participants were not always willing to supply this information, and partial postcode sufficient to capture derived information on deprivation may be appropriate in future studies. Our sample largely consists of white British women, therefore limiting the generalisability of our results to women of other ethnicities. Our sample also underrepresents less educated and more deprived women. Data on young women’s education, deprivation and ethnicity suggested that the acceptability of extra genital testing did not change with respect to education or deprivation levels, or whether participants’ identified ethnicity was ‘white’ or otherwise. Furthermore, Depcat [NHS Lothian Depcat) was used to measure deprivation in this study, as opposed to the more widely used Scottish Index of Multiple Deprivation (SIMD) [Scottish Government SIMD]. Both measures calculate deprivation differently, making the comparison between our sample and the population was challenging. Future studies should use SIMD. The main limitation of the qualitative phase of the study was that only women who were willing to return to the clinic and take the tests were interviewed. Although we invited women who did not want to do the testing to be interviewed by telephone none volunteered. Therefore, we are unable to identify factors that would prevent women taking the tests, beyond those provided in answers to the questionnaire.

## Conclusions

This feasibility study indicates promising acceptability of extra genital tract testing for *Chlamydia trachomatis*, especially for self-taken throat swabs. However, acceptability of ano-rectal testing appears to be related to age, with almost half of women under 20 reluctant to do this. The reasons behind this need to be addressed, given the high rates of *C*. trachomatis infection particularly in women under 20 years of age. The results of the study are broadly positive, and extragenital testing, particularly at the ano-rectal site may increase the accuracy of *C. trachomatis* diagnosis. Therefore, the results support future clinical trials that involve women self-sampling extragenital sites, to investigate if additional extra genital testing to routine chlamydia testing does result in the detection of more cases, and lead to the prevention of infective sequelae for women.

## Additional files

Additional file 1: Extragen Questionnaire. (PDF 224 kb)

Additional file 2: Interview topic guide. (DOCX 17 kb)

## Data Availability

The datasets used and/or analysed during the current study are available from the corresponding author on reasonable request.

## Abbreviations

C. trachomatis: Chlamydia trachomatis
MSM: Men who have sex with men
NAAT: Nucleic acid amplification test
PID: Pelvic inflammatory disease
SIMD: Scottish Index of Multiple Deprivation
STI: Sexually transmitted infection

## Footnotes

1 Tae dae – to do

2 Didnae – didn’t
