# Supplementary material for: Understanding the attitudes and acceptability of extra-genital Chlamydia testing in young women: evaluation of a feasibility study
Source: BMC Public Health. 2019 Jul 24;19:992. doi: 10.1186/s12889-019-7313-0 (PMC6657166; doi:10.1186/s12889-019-7313-0)
Supplement: Supplementary file 1 — Extragen Questionnaire. (PDF 224 kb) [file 12889_2019_7313_MOESM1_ESM.pdf]

E
x
t
r
a
g
e
n

Questionnaire

Please complete all questions as fully as you can.

By completing the questionnaire, you are indicating that you agree to participate and are giving your consent for the information you provide to be used in this research study. There is no way that the completed questionnaire can be traced back to you.

Please remember that you don't have to complete this questionnaire (or any question in it) if you don't want to - this is entirely your choice.

SECTION 1: Your feelings about the proposed testing

Please remember that your responses are confidential and your answers will not affect your treatment today

1	In future, if **you** thought you would get MORE ACCURATE RESULTS by taking a swab your throat (i.e (taking a test using a cotton bud that is wiped to the back of your throat) was also included as part of Chlamydia testing, would you be happy to DO THIS **YOURSELF**? (Please tick one box only)

Yes ☐ 1 No ☐ 2 Unsure ☐ 3

2	If no then please state why (tick as many as apply)

- I worry about taking the sample from the wrong place ☐ 1
- I prefer a health care professional to take the sample ☐ 2
- I don’t want to put any thing in my throat ☐ 3
- Other - please tell us your reason (Please write below)

____

Throat sampling

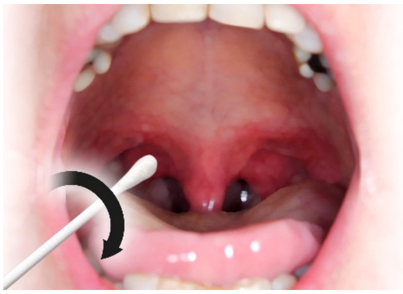

Taking the swab

- Open your mouth wide and wipe the swab around the tonsil area on either side of your throat several times

Images reproduced with permission NHS Lothian

3	In future, if ***you*** thought you would get MORE ACCURATE RESULTS by taking a sample from the entrance to your back passage (anus) was also included as part of Chlamydia testing, would you be happy to DO THIS **YOURSELF** ? (Please tick one box only)
	Yes ☐ 1 No ☐ 2 Unsure ☐ 3
4	If no then please state why (tick as many as apply)

- I worry about taking the sample from the wrong place ☐ 1
- I dont like the idea of touching my own anus ☐ 2
- I prefer a health care professional to take the sample ☐ 3
- I dont want to put any thing in my anus ☐ 4
- Other - please tell us your reason (Please write below)

____

Anal sampling

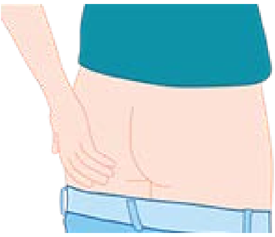

Parting buttocks

- Part your buttocks or squat to take the test

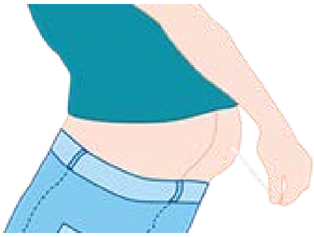

Inserting the swab

- Insert the swab 3-4 cm

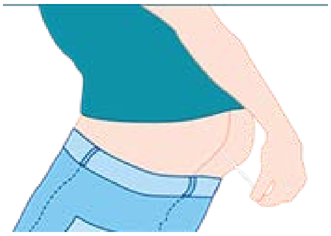

Taking the swab

- Gently twist the swab and move from side to side

Images reproduced with permission from Sandyford Services, NHS Greater Glasgow and Clyde

SECTION 2: Information about your sexual history

Please remember that your responses are confidential and this information cannot be used to identify you

5 How many sexual partners have you had in the previous 6 months? Please **tick** one box only

0 ☐ 1   1 ☐ 2   2-3 ☐ 3   More than 3 ☐ 4

6 Do you consider yourself to be? Please **tick one box only**

Heterosexual or straight ☐ 1   Gay or Lesbian ☐ 2

Bisexual ☐ 3   Prefer not to say ☐ 4

Prefer to self describe ____________________ 5

7 Have you ever had any of these forms of sexual activity? Please **tick all** boxes that apply

Vaginal ☐ 1   Oral ☐ 2

Anal ☐ 3   None of these ☐ 4

8 Do you use condoms as a method of contraception? Please **tick** one box only

Never ☐ 1   Rarely ☐ 2   Mostly ☐ 3   Always ☐ 4

9 What method of contraception do you normally use? Please **tick all** boxes which apply

The Pill ☐ 1   Implant (e.g.Nexplanon) ☐ 2

Male condoms ☐ 3   Contraceptive patch (e.g. Evra) ☐ 4

Copper Coil/intrauterine device ☐ 5   Female condom ☐ 6

Injections (e.g. Depo Provera or Sayana ☐ 7   Cap/diaphragm ☐ 8

Partner has been sterilised (vasectomy) ☐ 9   I have been sterilised ☐ 10

I do not use any method ☐ 11

Other method of protection (please say what) ____________________

10	Have you ever tested positive for an Sexually transmitted infection? Please **tick** one box only

Yes ☐ 1   No ☐ 2   Unsure ☐ 3

11	If you have ever had a positive STI diagnosis? Please **tick all** boxes which apply

Chlamydia ☐ 1   HIV ☐ 2

Gonorrhoea ☐ 3   Herpes ☐ 4

Syphilis ☐ 5   Warts ☐ 6

Other (please say what) ________________________________

SECTION 3: Information about yourself

Please remember that your responses are confidential and this information cannot be used to identify you

12	What age are you (in years)?

Please write in ☐

13	What are the first five characters of your home postcode? If do you not know your postcode please indicate the area you live e.g. Gorgie (Please **write** in)

E	H				

e.g. E H 1 4 8

14	Do you smoke? (Please tick **one box only**)

Yes ☐ 1   No ☐ 2   Ex-smoker ☐ 3

15	What is you highest level of education to date? (Please tick **one box only**)

No educational qualifications ☐ 1

Standard grades, intermediate 1 or 2, GCE/GCSEs ☐ 2

Higher, Advance Higher, A Levels ☐ 3

Vocational qualification (e.g. Access, SVQ, SCOTVEC, BTEC) ☐ 4

Higher National Certificate (HNC)/ Diploma (HND) ☐ 5

16	Which of the following best describes your ethnic background?<br>(Please tick **one box only**)

White (Scottish, British, Irish or any other White background) ☐ 1

Asian or Asian British (Bangladeshi, Indian, Pakistani or any other Asian background) ☐ 2

Black or Black British (African, Caribbean or any other Black background) ☐ 3

Chinese or Chinese British ☐ 4

Mixed or any other ethnic background (Please write in below) ☐ 5

____

You have now reached the end of the questionnaire.
Please place the questionnaire in the box at reception

If you wish to be contacted as part of the interview part of the study please fill in the form on the next page

Many thanks for your time and help

Edinburgh Napier UNIVERSITY

NHS Lothian

CHIEF SCIENTIST OFFICE

SECTION 4: Consent for interview

Please remember that participation is voluntary and you do not need to provide your contact details unless you wish to take part in the interview part of the study

Contact Details for Interview:

First name ………………………… Surname……………………

Telephone number ………………………………………………………………

Email ………………………………………………………………

Address (Number, street) ………………………………………………………………

City …………………………… Postcode ……………………..

I prefer to be contacted by (please tick):

PHONE ☐ EMAIL ☐ POST ☐

ANY OF ABOVE
I prefer to be contacted (please tick all that apply):

	Any time	Morning (9am-12 noon)	Afternoon (12 noon-5pm)	Evening (5-8pm)
Weekday	☐	☐	☐	☐
Weekend	☐	☐	☐	☐
Any other specific instructions:				

Please detach this sheet, seal it in the envelope provided and hand it in to reception
