# Supplementary material for: Understanding the attitudes and acceptability of extra-genital Chlamydia testing in young women: evaluation of a feasibility study
Source: BMC Public Health. 2019 Jul 24;19:992. doi: 10.1186/s12889-019-7313-0 (PMC6657166; doi:10.1186/s12889-019-7313-0)
Supplement: Supplementary file 2 — Interview topic guide. (DOCX 17 kb) [file 12889_2019_7313_MOESM2_ESM.docx]

**ExtraGen study**

**Interview topic guide – face to face interviews.**

Thank participant for attending; make sure they have read the PIS and understand the study, and give them the opportunity to ask questions. Complete the consent form.

Before you go to do the tests, I’d like to ask you what you think about the tests. How do you feel about doing the throat swab? How do you feel about doing the anal swab? Do you feel happy that you know what to do? What do you know about why we’re asking people to do tests this way?

*Give tests and instructions; research nurse takes them off to do the test.*

*Participant returns*.

How was that? How did you feel about doing the throat test? How did you feel about doing the rectal test? Better or worse than you’d imagined? Did you feel comfortable doing the tests?

Where the instructions sufficient for each test? (If not, why not?)

Is there anything that could be done to make the procedure better? More comfortable?

What other tests and checks have you had? (blood tests, cervical smear, any others)

How did you feel about them?

Do you think they are different to the throat and anal tests? Why?

How would you feel about being able to do these tests at home and post the swabs in for testing?

Is there anything else you’d like to say about the tests, or your experience at the clinic?

*Explain that swabs will be sent for testing; if a positive result comes back, they will be offered treatment.*

Thank you for taking part in the study.

**Interview topic guide – telephone interviews.**

Thank participant for taking part; make sure they have read the PIS and understand the study, and give them the opportunity to ask questions. Explain that the call will be recorded if they agree. Complete the consent form verbally (recorded).

As I don’t have your questionnaire in front of me, could you remind me, did you say you wouldn’t like to do the throat swab yourself? And did you say you wouldn’t like to do the rectal one?

Thinking about the throat swab, what was it that made you not want to do it? (explore further depending on their answers: possibility of gagging, don’t like the thought of it, prefer tests to be done by a nurse)

Thinking about the anal swab, what was it that made you not want to do that one? (explore further depending on their answers: don’t like the thought of it, don’t want to touch that part of their body, prefer tests to be done by a nurse)

What other tests and checks have you had? (blood tests, cervical smear, any others)

How did you feel about them?

Do you think they are different to the throat and anal tests? Why?

Would it make a difference if we discovered that test results are more reliable done this way?

Is there anything we could do differently in the clinic that might make you feel you could do the throat or the rectal test yourself?

How would you feel about being able to do these tests at home and post the swabs in for testing? Would you feel you could do them then? (Both, either?)

Is there anything else you’d like to say about the tests, or your experience at the clinic?

Thank you for taking part in the study.
